# Sonic Hedgehog Promotes Tumor Cell Survival by Inhibiting CDON Pro-Apoptotic Activity

**DOI:** 10.1371/journal.pbio.1001623

**Published:** 2013-08-06

**Authors:** Céline Delloye-Bourgeois, Benjamin Gibert, Nicolas Rama, Jean-Guy Delcros, Nicolas Gadot, Jean-Yves Scoazec, Robert Krauss, Agnès Bernet, Patrick Mehlen

**Affiliations:** 1 Apoptosis, Cancer and Development Laboratory–Equipe labellisée ‘La Ligue’, LabEx DEVweCAN, Centre de Cancérologie de Lyon, Institut National de la Santé et de la Recherche Médicale (INSERM) U1052– Centre National de la Recherche Scientifique (CNRS) Unité Mixte de Recherche (UMR5286), Université de Lyon, Centre Léon Bérard, 69008 Lyon, France; 2 Endocrine Differentiation Laboratory, Centre de Cancérologie de Lyon, INSERM U1052–CNRS UMR5286, Université de Lyon, Hospices Civils de Lyon, Hôpital Edouard Herriot, Anatomie Pathologique, 69437 Lyon, France; 3 Department of Developmental and Regenerative Biology, Mount Sinai School of Medicine, New York, New York, United States of America; Stanford University, United States of America

## Abstract

The Hedgehog signaling is a determinant pathway for tumor progression. However, while inhibition of the Hedgehog canonical pathway—Patched–Smoothened–Gli—has proved efficient in human tumors with activating mutations in this pathway, recent clinical data have failed to show any benefit in other cancers, even though Sonic Hedgehog (SHH) expression is detected in these cancers. Cell-adhesion molecule-related/down-regulated by Oncogenes (CDON), a positive regulator of skeletal muscle development, was recently identified as a receptor for SHH. We show here that CDON behaves as a SHH dependence receptor: it actively triggers apoptosis in the absence of SHH. The pro-apoptotic activity of unbound CDON requires a proteolytic cleavage in its intracellular domain, allowing the recruitment and activation of caspase-9. We show that by inducing apoptosis in settings of SHH limitation, CDON expression constrains tumor progression, and as such, decreased CDON expression observed in a large fraction of human colorectal cancer is associated in mice with intestinal tumor progression. Reciprocally, we propose that the SHH expression, detected in human cancers and previously considered as a mechanism for activation of the canonical pathway in an autocrine or paracrine manner, actually provides a selective tumor growth advantage by blocking CDON-induced apoptosis. In support of this notion, we present the preclinical demonstration that interference with the SHH–CDON interaction triggers a CDON-dependent apoptosis *in vitro* and tumor growth inhibition *in vivo*. The latter observation qualifies CDON as a relevant alternative target for anticancer therapy in SHH-expressing tumors.

## Abstract 2

CDON is a novel Sonic Hedgehog (SHH) dependence receptor and targeting the SHH-CDON interaction could represent an alternative therapeutic strategy for patients suffering from tumors that express high SHH levels.

## Author Summary

Abnormal activity of the Hedgehog signaling pathway has been linked with tumor progression; deregulation of this pathway can occur in cancers either by mutations in key effectors of the so-called canonical signaling pathway or by aberrant expression of Hedgehog itself. While inhibition of the canonical signaling pathway has proved efficient for treating some cases of human tumors in which the Hedgehog pathway was shown to be overactive, recent clinical data have shown this approach is not often successful. We have identified a new role for the Hedgehog ligand that we propose can explain why inhibiting the canonical signaling pathway generically doesn't always produce the desired therapeutic effect. We show that Hedgehog expression provides a selective growth advantage for tumours. It does so by binding to a novel dependence receptor called Cell adhesion molecule-related, down-regulated by oncogenes (CDON). This type of receptor depends on the presence of its ligand, in the absence of which it induces apoptosis. We show that interfering with the Hedgehog–CDON interaction triggers CDON-dependent apoptosis *in vitro*, and tumor growth inhibition *in vivo*. Development of a candidate drug interfering with Hedgehog–CDON interaction could be of potential benefit to the large number of patients suffering from cancers with high SHH levels, but not currently responding to therapies aimed at antagonizing the Hedgehog signaling pathway.

## Introduction

Sonic Hedgehog (SHH) is a key secreted morphogen with multiple known functions, both during embryonic development and throughout adulthood. Canonical SHH signaling is mediated mainly via its interaction with the receptor Patched 1 (Ptc). Binding of SHH to Ptc relieves its suppressive effect on Smoothened (Smo), an orphan seven-transmembrane receptor that initiates a signaling pathway leading to the activation of the glioma-associated (Gli) family of transcription factors. SHH and its downstream signaling are known to regulate many developmental processes including ventrodorsal patterning of the neural tube, establishment of limb polarity, and development of the foregut and axio-cranial skeleton [1],[2]. In adults, SHH signaling is mainly quiescent, being physiologically reactivated only during specific processes such as tissue maintenance and repair. However, SHH signaling appears to be crucial during tumor progression [3]–[6]. Abnormal induction of SHH signaling through different means—e.g., down-regulation or mutation of SHH receptor(s) or effectors, autocrine, or paracrine expression of SHH—has been associated with many different types of human cancers [7]–[10]. As a consequence, SHH and its downstream signaling effectors are considered as potentially important targets for anticancer strategies [11],[12].

**Figure 1:**
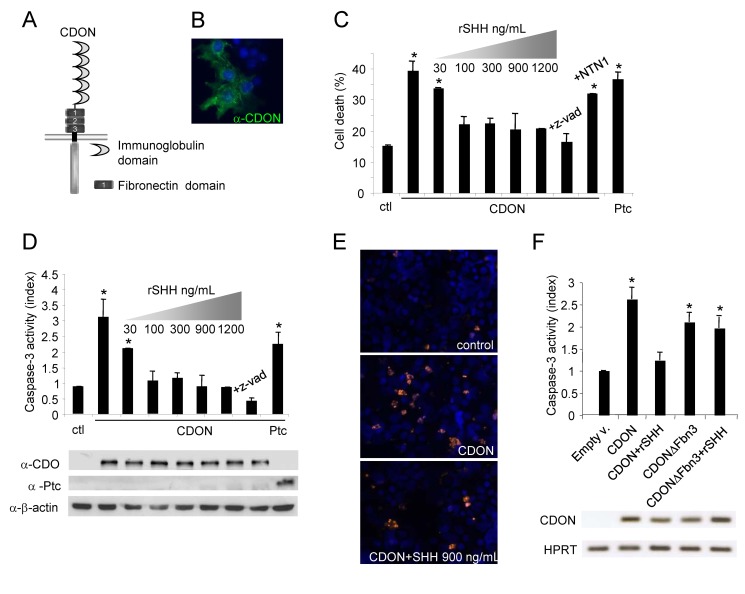
CDON induces apoptosis in the absence of SHH. (A) Schematic representation of CDON protein structural domains. (B) CDON immunofluorescence staining of HEK293T transfected with a full-length mouse CDON expression plasmid. CDON (green) and nuclei (Hoechst in blue) staining are shown. (C–E) Cell death induction in HEK293T cells was quantified by trypan blue exclusion assay (C), caspase-3 activity assay (D), or TUNEL staining (E) after transfection with mock, CDON, or Ptc expression vectors. Increasing amounts of recombinant SHH added in the culture medium are indicated. Recombinant netrin–1 (NTN1) was added in the culture medium as a negative control. The general caspase inhibitor z-VAD-fmk was used to block caspase-dependent cell death. In (D), lower panel shows detection of CDON, Ptc, and β-actin proteins by Western Blot. In (E), TUNEL-stained cells (orange) and nuclei (Hoechst staining in blue) are shown. (F) Apoptotic cell death induction as measured by caspase-3 activity was quantified in HEK293T cells transfected either with full-length rat CDON or with rat CDON deleted from its SHH-binding site (CDONΔFbn3) alone or with recombinant SHH (rSHH) added in excess in the culture medium. Lower panel shows PCR products obtained after amplification with rat CDON and human HPRT-specific primers. For (B–D), F error bars indicate s.d. Statistical treatment of the data was performed using a two-sided Mann–Whitney test compared to mock-transfected condition (*p<0.05; **p<0.01).

CDON (or CDO) and Brother of CDON (BOC), two homologous members of the Neural Cell Adhesion Molecule (N-CAM) family (see graphic representations in Figure 1A and Figure S1A), were recently described as receptors for SHH, and hypothetical co-receptors of Ptc, that positively regulate the SHH signaling pathway [13]–[17]. CDON resembles in many aspects DCC (Deleted in Colorectal Cancer), the prototypical netrin-1 dependence receptor. Dependence receptors share the property of creating a cellular state of dependence upon their ligands by inducing apoptosis when unbound by their respective ligands [18]–[20]. Such a dependence effect is thought to be crucial to eliminate tumor cells that would otherwise develop in settings of ligand unavailability [21]–[23]. These dependence receptors now include more than 17 different transmembrane receptors [24], and Ptc was recently described as a dependence receptor that triggers apoptosis via the recruitment of a caspase-activating complex called the dependosome [25],[26]. We investigated here whether CDON may, similarly to Ptc, behave as a SHH dependence receptor, and as such display a role in regulating cell survival.

## Results

### CDON Induces Apoptosis in the Absence of SHH

To determine whether CDON and/or BOC (see graphic representation in Figure 1A and Figure S1A) are dependence receptors, HEK293T and NIH-3T3 cells were transiently transfected with expression constructs for both receptors. Expression of CDON and BOC could be detected at the plasma membrane (Figure 1B and not shown). While, as observed for Ptc, CDON expression was associated with increased cell death as measured by a trypan blue exclusion assay (Figure 1C and not shown), no significant effect was observed with BOC expression (Figure S1B). CDON-induced cell death was, at least in part, apoptotic, since it was associated with a specific increase in caspase-3 activity (Figure 1D and Figure S1B) and DNA-fragmentation as measured by TUNEL (Terminal deoxynucleotidyl transferase–mediated deoxyUridine triphosphate Nick End Labeling) staining (Figure 1E). Moreover, such a pro-apoptotic effect was caspase-dependent since the addition of the general caspase inhibitor z-VAD-fmk inhibited CDON-induced cell death (Figure 1CD).

We therefore investigated whether, as expected for a dependence receptor, addition of the ligand could prevent CDON-induced cell death. As shown in Figure 1CD and Figure S1C, cell death associated with CDON expression was inhibited in a dose-dependent manner by addition of exogenous recombinant SHH. Treatment with recombinant netrin-1, the ligand of the dependence receptor DCC, had no significant incidence on CDON-associated-cell death (Figure 1C), while the addition of SHH had no effect on DCC-induced apoptosis (Figure S1D). Not only SHH but also desert hedgehog inhibited CDON-induced cell death (Figure S1E). Moreover, deletion of the third fibronectin domain of CDON, which mediates its interaction with SHH, turned CDON into a constitutive pro-apoptotic mutant, unresponsive to SHH (Figure 1F). Altogether, these data demonstrate that CDON behaves as a SHH dependence receptor *in vitro*. Preliminary data support the view that SHH and CDON pro-apoptotic activity are also important *in vivo* during the development of the first branchial arch [27].

### CDON Triggers Apoptosis Via a Caspase-9–Dependent Mechanism

**Figure 2:**
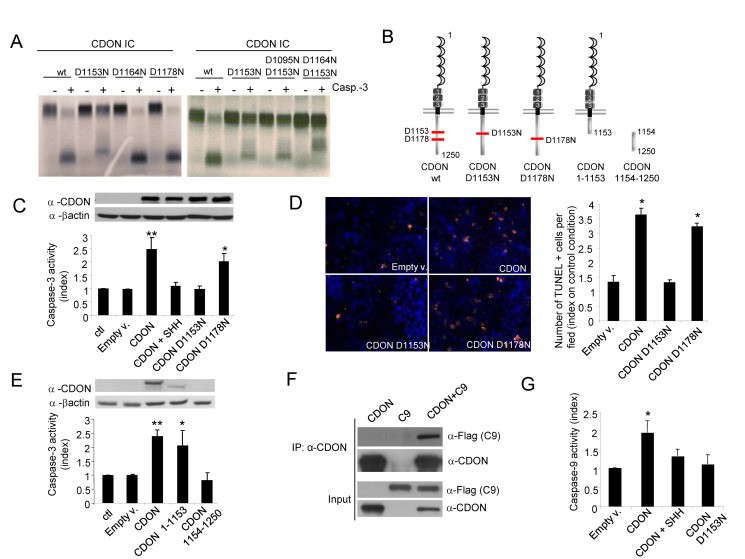
CDON triggers apoptosis through CDON proteolytic cleavage and recruitment and activation of caspase-9. (A) *In vitro*–translated CDON intracellular domain (CDON-IC) wild-type (wt) or mutated on one (left panel) or two (right panel) aspartic acid residues were incubated in the absence or in the presence of recombinant purified active caspase-3. Autoradiographs show the cleavage by caspase-3 of CDON-IC wt, whereas CDON-IC–D1153N was weakly cleaved and CDON-IC–D1153N–D1164N was almost completely resistant to cleavage. The band appearing in the CDON D1164N–D1153N probably represents a cryptic site. (B) Schematic representation of CDON and its different mutant constructs. CDON-main (D1153) and secondary (D1178) caspase cleavage sites are shown. (C–D) Apoptotic cell death induction as measured by caspase-3 activity (C) was quantified in HEK293T cells transfected with wild-type (CDON) full-length or mutated full-length CDON expression plasmids and by TUNEL (D) staining was quantified in HEK293T cells transfected with wild-type (CDON) full-length or mutated full-length CDON expression plasmids. Quantifications of TUNEL positive cells (orange) and nuclei (Hoechst in blue) are shown. (E) Apoptotic cell death induction as measured by caspase-3 activity was quantified in HEK293T cells transfected with constructs encoding full-length CDON or the CDON hypothetical fragments resulting from its cleavage by caspase at D1153 (CDON1–1153 and CDON 1154–1250). (F) HEK293T cells were transfected with constructs encoding CDON and/or caspase-9 and cell lysates were subjected to immunoprecipitation with a CDON-specific antibody. CDON and caspase-9 proteins were detected by Western blot in immunoprecipitated and input fractions. (G) Caspase-9 activity was quantified in HEK293T cells transfected with wild-type (CDON) full-length or mutated full-length CDON (D1153N) expression plasmids. For (C–F) and (G), data are means of at least three independent assays. Error bars indicate s.d.

One common feature of most dependence receptors identified to date is the cleavage of their intracellular domain by caspases, a preliminary step required for their pro-apoptotic activity. We thus investigated whether CDON shares this property *in vitro*. As shown in Figure 2A, the CDON intracellular domain (CDON-IC, amino acids 985 to 1250) was cleaved *in vitro* when incubated with purified active caspase-3, suggesting the existence of at least one cleavage site in CDON-IC. To determine the putative caspase cleavage site, aspartic acid residues in CDON intracellular domain were successively mutated. While the Asp^1178^ to Asn mutation (CDON-IC D1178N) had no effect on CDON-IC cleavage, mutation of Asp^1153^ to Asn (CDON-IC D1153N) strongly inhibited cleavage by caspase (Figure 2A, left panel). However, the Asp^1153^ to Asn mutation revealed the presence of a secondary caspase cleavage site in CDON-IC, since the incubation of the CDON-IC–D1153N with active caspase-3 was associated with the presence of another faint band that indicated an additional cleavage fragment. We therefore mutated different Asp residues in CDON-IC–D1153N and assessed cleavage by caspase-3. As shown in Figure 2A (right panel), the Asp^1153^/Asp^1164^ to Asn double mutation almost completely suppressed caspase-3 cleavage, indicating that CDON was cleaved *in vitro* by caspase at Asp^1153^ and Asp^1164^, with Asp^1153^ being the main cleavage site (Figure 2A). Even though, similarly to many other dependence receptors, we failed to detect caspase cleavage of CDON in cell culture, full-length CDON and its putative cleavage fragment were detected from E12.5 mouse embryos extracts and the incubation of these extracts with z-VAD-fmk decreased the presence of the cleavage fragment, hence supporting the view that CDON is cleaved by caspase *in vivo* (Figure S1F).

To evaluate the functional relevance of CDON-IC cleavage, the full-length CDON D1153N and CDON D1178N (noncaspase cleavage site mutation used as a control) mutants were transiently expressed in HEK293T and NIH3T3 cells, and cell death was assessed by measuring caspase-3 activity and DNA fragmentation (TUNEL staining). Whereas wild-type CDON and CDON D1178N both triggered apoptotic cell death, CDON D1153N mutant and the CDON D1153N/D1164N double mutant failed to induce any increase in caspase-3 activity or DNA fragmentation (Figure 2B–D and unpublished data). Thus, the cleavage of CDON's intracellular domain at Asp^1153^ is a prerequisite for its pro-apoptotic activity.

In an attempt to identify the region of CDON implicated in cell death induction, the pro-apoptotic activity of the CDON fragment potentially released by the caspase cleavage (CDON1154–1250) or the remaining membrane-bound CDON truncated at Asp^1153^ (CDON1–1153) was assessed upon expression in HEK293T (Figure 2E and Figure S2A). As shown in Figure 2E, the expression of the CDON1–1153 fragment triggered an increase in caspase-3 activation similar to that of full-length CDON, whereas the CDON1154–1250 fragment had no effect. Moreover, while SHH addition in the medium suppressed CDON-induced apoptosis, it had no effect on CDON1–1153–induced apoptosis (Figure S2B). Together these results support the view that, in the absence of SHH, CDON is cleaved by caspase at Asp^1153^, leading to the exposure of a pro-apoptotic domain located in the N-terminal intracellular region (residues 985 to 1153).

Recent data have suggested that, once cleaved by an active caspase, at least some dependence receptors recruit a caspase-activating complex named the dependosome, which includes the apical caspase-9 [26],[28]. We thus investigated whether CDON could recruit and activate caspase-9. As shown in Figure 2F, in the absence of SHH, CDON efficiently pulled down caspase-9 when co-expressed in HEK293T cells. Similar interaction of CDON with caspase-9 can be detected with endogenous proteins (Figure S2C). CDON-induced apoptosis is dependent on caspase-9 but not on caspase-8, since a caspase-9 dominant negative mutant efficiently blocked CDON-induced apoptosis, while a caspase-8 dominant negative mutant did not (Figure S2D). Moreover, we show that CDON triggered caspase-9 activation, which was blocked upon SHH treatment, whereas CDON mutated at its caspase cleavage site did not have such an effect (Figure 2G). Together, these data support a model in which, in the absence of SHH, CDON is initially cleaved by a first set of active caspases (or other proteases), leading to the exposure of a pro-apoptotic domain that recruits and activates caspase-9, probably through a caspase-activating complex that remains to be identified.

Because CDON has been shown to participate in the Ptc–Smo–Gli canonical pathway when engaged by SHH [13], and because Ptc has been shown to trigger apoptosis through caspase-9 in the absence of SHH [26], we investigated possible cross-talks between CDON-induced apoptosis and Ptc-mediated signalings. We first analyzed whether enforced expression of CDON alters the gene transcription response triggered by the Smo–Gli pathway. Neither CDON expression nor the expression of various CDON mutants presented above triggered transactivation of a Gli-1 reporter gene, or affected Gli-1 reporter gene activation mediated by Gli-1 activation (induced through transfection of Gli-1 itself or through treatment with the Smo agonist SAG) (Figure S3A). Reciprocally, apoptosis induced by enforced CDON expression was affected neither by activation of the Smo–Gli-1 pathway (induced through transfection of Gli-1 or through treatment with SAG, Figure S3B and Figure S1C) nor by inhibition of this pathway through treatment with the Smo antagonist cyclopamine (not shown). We next investigated whether Ptc pro-apoptotic activity is implicated in CDON-mediated apoptosis. As also observed for other dependence receptors—e.g., DCC, UNC5H, or TrkC—the intracellular domain of Ptc mutated at its caspase cleavage site (Ptc-7IC–D1392N, here called Ptc-DN) functions as a dominant negative for the pro-apoptotic activity of endogenous Ptc [25],[29],[30]. Ptc-DN, while efficiently blocking Ptc-induced apoptosis, did not affect CDON-induced apoptosis in HEK293T cells (Figure S3C). Similar effect was seen *in vivo* during branchial arch formation as Ptc-DN failed to block CDON-mediated neural crest cell apoptosis observed upon SHH titration [27]. Reversely, Ptc-induced apoptosis was not inhibited by CDON-IC–D1153N/D1164N, while this construct efficiently blocked CDON-induced apoptosis (Figure S3D). Similar results were obtained through silencing by siRNA approach as CDON-induced apoptosis was not affected by Ptc silencing and reversely Ptc-induced apoptosis was not inhibited by CDON silencing (Figure S3E). Thus, the death signal induced by unbound CDON is independent of Ptc pro-apoptotic activity and does not involve the canonical Ptc–Smo–Gli pathway, but is dependent on caspase-9 recruitment and activation.

### CDON Expression Is a Constraint for Tumor Progression

It has been suggested that the pro-apoptotic activity of unbound dependence receptors is a mechanism for eliminating tumor cells that would otherwise proliferate in an environment with limited ligand availability or migrate to tissues devoid of ligand during metastatic dissemination [21],[22],[24]. As such, dependence receptors behave as tumor suppressors. Along this line, genetically engineered mouse bearing a caspase mutant site in DCC developed spontaneously colorectal cancer [23]. In agreement with this view, dependence receptor expression is often reduced or completely lost in various human cancers [31],[32].

**Figure 3:**
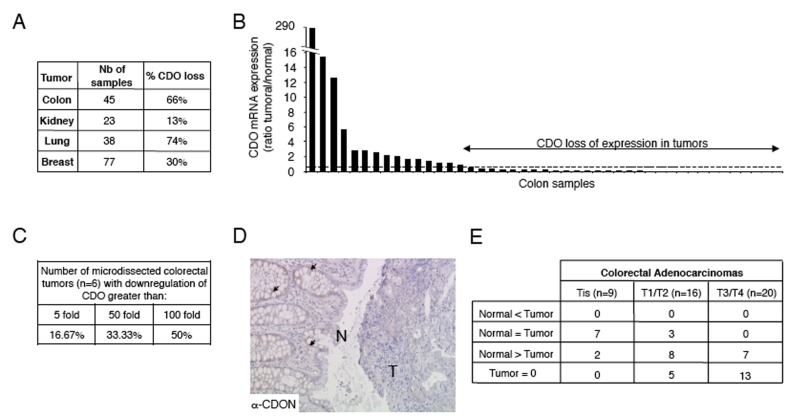
CDON expression is decreased in human cancers. (A) Quantification of CDON expression by Q-RT-PCR in a panel of 183 human tumors. For lung, colon, and kidney, each tumor sample was compared to a normal paired tissue. CDON expression in breast tumors was compared to the median CDON expression level in the 77 samples. For each type of cancer, the percentage of tumors showing loss of CDON expression is indicated. Loss of CDON expression in the tumor is considered when more than a 2-fold decrease of expression is observed as compared to the normal tissue (lung, colon, kidney) or to the median CDON expression value (breast). (B) Quantification of CDON expression by Q-RT-PCR in a panel of 45 human colorectal tumors and paired normal tissues. Data are presented as a ratio of CDON expression between tumor and normal paired tissue for each patient. Loss of CDON expression is considered when more than a 2-fold decrease of expression in the tumor is observed (indicated by the dashed line). (C) Quantification by Q-RT-PCR of CDON expression between normal and tumor tissue in human colon samples after laser capture microdissection. (D) Representative CDON immunohistochemistry on a human colon adenocarcinoma. N, normal tissue; T, tumor tissue. Note that CDON is clearly detected in the normal tissue (arrows), whereas the tumor tissue is only weakly, if not, stained. (E) Table recapitulating CDON protein level (quantified by immunohistochemistry) in nine isolated (Tis), 16 stage 1/2 (T1/2), and 20 stage 3/4 (T3/4) human colorectal adenocarcinomas according to the TNM classification. The number of cases showing either a higher, an equal, a lower, or no CDON expression in the tumor as compared to the normal tissue is indicated.

To assess whether CDON expression is generally affected in human cancers, we then analyzed CDON expression in a panel of human tumors and their corresponding normal tissues by Q-RT-PCR. As shown in Figure 3AB and Figure S4A, CDON expression was decreased in a large fraction of colorectal and lung cancers and in a sizeable fraction of kidney and breast cancers. Similar reduction of CDON expression was observed by dot blot array analysis in breast, ovarian, uterine, and thyroid cancers (Figure S4B). In addition, laser capture microdissection on colorectal tumors versus normal adjacent tissues followed by Q-RT-PCR was performed on six colon cancer samples and showed in most cases a massive decrease of CDON expression in tumor cells (Figure 3C). Consistent with a specific decreased expression in tumor cells, CDON immunohistochemistry in a panel of 45 human colon adenocarcinomas showed a marked decrease of CDON expression in epithelial tumor cells as compared to adjacent normal tissue (Figure 3DE). Interestingly, in this panel of colorectal adenocarcinomas, CDON expression is inversely correlated with tumor grade according to the TNM classification (Figure 3E, χ^2^ test: *p* = 4.3^E-5^). Together this suggests that CDON expression is a constraint for tumor progression in the human pathology.

**Figure 4:**
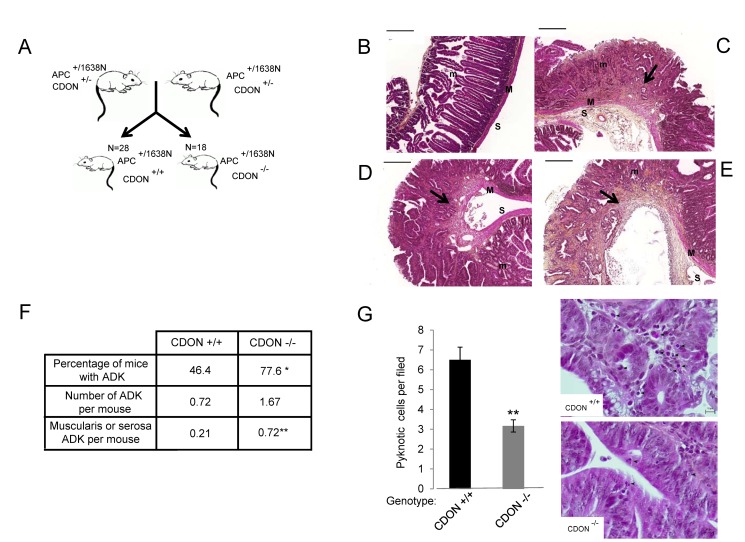
CDON is a *bona fide* tumor suppressor. (A) Cohorts of CDON^−/−^ APC^+/1638N^ mice were generated to analyze the effect of *CDON* genetic invalidation on intestinal tumorigenesis compared to CDON^+/+^ APC^+/1638N^. (B–E) Representative images of Haematoxylin–Eosin–Saffron (HES) staining of normal intestinal epithelium (B) compared to adenocarcinomas with mucosa (C) muscularis (D) or serosa (E) local invasion observed in CDON^−/−^ APC^+/1638N^ mice. m, mucosa; M, muscularis; S, serosa; scale bar, 200 µm. (F) Frequency and incidence of adenocarcinomas (ADK) in CDON^+/+^ APC^+/1638N^ mice (*n* = 28) compared to CDON^−/−^ APC^1638N^ mice (*n* = 18). Tumour classification was performed according to international recommendations (χ^2^ test; *p<0.05 and Student *t* test; **p<0.01). (G) Cell death was quantified in high grade adenomas from three mice of each genotype. Representative images of pyknotic cells from HES CDON^+/+^ APC^+/1638N^ mice compared to CDON^−/−^ APC^+/1638N^ mice are shown in right panels. Scale bar, 10 µm. Error bars indicate s.e.m. Statistical treatment of the data was performed using a two-sided Mann–Whitney test (**p<0.01).

We thus analyzed whether the decreased expression of CDON seen in a fraction of human colorectal cancers has a causal implication in tumor progression. To do so, we investigated the possibility that CDON inactivation in mice may affect tumour progression by analyzing the effect of the CDON mutation on adenocarcinoma (ADK) formation in an APC^+/1638N^ genetic background (Figure 4A). APC is a well-known tumour suppressor gene in human colorectal cancer, and APC mutations in mice are associated with neoplasm formation. We chose the APC^+/1638N^ mutant mice, which were shown to develop tumours in the intestinal tract at a moderate level [33]. Consistent with previous reports, the number of adenocarcinomatous lesions per CDON^+/+^APC^+/1638N^ control mice was 0.72 (Figure 4B–F). The incidence of adenocarcinomas was increased by more than 2.3-fold in CDON^−/−^ APC^+/1638N^ mice (Figure 4B–F). Moreover, whereas adenocarcinomas were detected in 46.4% of the CDON^+/+^APC^+/1638N^ control mice, consistent with previous reports [33], the frequency of CDON^−/−^APC^+/1638N^ mice with adenocarcinomas was markedly increased to 77.6% (Figure 4F; *p*<0.05). Of interest, CDON^−/−^APC^+/1638N^ mice showed over 3.4-fold more of aggressive adenocarcinomas with muscularis or serosal invasion compared to CDON^+/+^APC^+/1638N^ controls (Figure 4B–F; *p*<0.01).

We next investigated whether CDON loss, as expected according to the pro-apoptotic activity of CDON, may enhance tumour cell survival at the transition from adenoma to adenocarcinoma. We therefore assessed whether apoptosis is quantitatively different in low-grade tumors of CDON^−/−^APC^+/1638N^ versus CDON^+/+^APC^+/1638N^ control mice. As shown in Figures 4G and S4C, we observed a marked decrease in apoptosis rate in high-grade adenomas from CDON^−/−^APC^+/1638N^ mice compared to that in size-matched CDON^+/+^APC^+/1638N^ controls. Thus together with the loss of expression of CDON in a fraction of human cancers and with the fact that high-throughput sequencing consortia have reported the presence of a large number of sprayed missense mutations in the coding sequence of CDON in human cancers (see http://cancer.sanger.ac.uk/cosmic/gene/overview?ln=CDONN), these data argue that CDON is *a bona fide* tumor suppressor probably thanks to its dependence receptor function.

### Targeting SHH Inhibits Tumor Growth Via CDON-Induced Apoptosis

The therapeutic relevance of CDON as a negative regulator of tumor growth is obviously minimal in the fraction of cancers with down-regulation of CDON. However, according to the mode of action of dependence receptors, it could be speculated that, rather than a loss of CDON expression, some tumors may have selected for up-regulation of SHH, which should similarly be associated with a constitutive inactivation of CDON-induced cell death. In agreement with this hypothesis, autocrine or paracrine expression of SHH has been extensively described in different human cancers [4],[6],[10],[11],[34]–[37]. The general view is that this autocrine or paracrine SHH expression is a mechanism that constitutively activates Ptc-Smo signaling either in tumor cells or more generally in stromal cells [10],[11]. According to the newly identified pro-apoptotic function of CDON in settings of SHH limitation, we thus investigated whether in SHH-expressing tumors, SHH does not (solely) activate the canonical signaling in stromal cells but also (or rather) blocks CDON-mediated tumor cell death.

**Figure 5:**
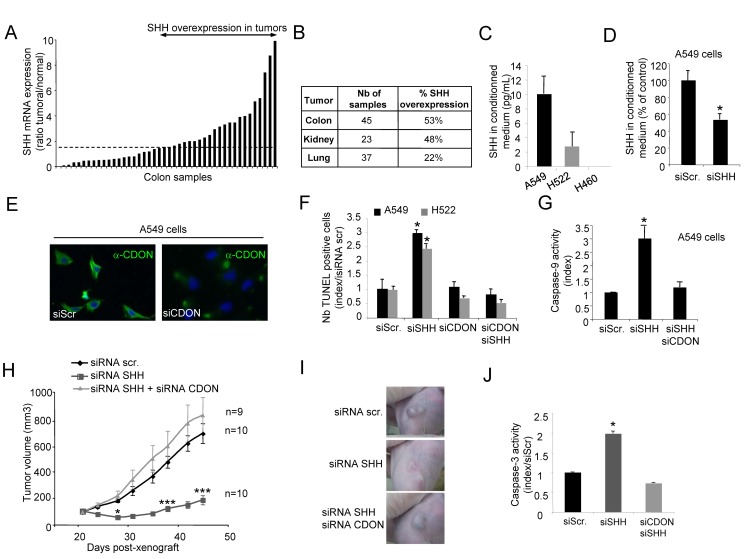
Inhibition of SHH triggers cancer cell death *via* CDON-induced apoptosis. (A) Quantification of SHH expression by Q-RT-PCR in a panel of 45 human colorectal tumors and paired normal tissues. Data are presented as a ratio of SHH expression between tumor and normal tissue for each sample. Overexpression of SHH is considered when more than a 1.5-fold increase in the tumor is observed (indicated by the dashed line). (B) Quantification of SHH expression by Q-RT-PCR in a panel of 105 human tumors. Each tumor sample was compared to a normal paired tissue. For each type of tissue, the percentage of tumors showing an increase in SHH (considered when a more than 1.5-fold increase in expression is observed as compared to the normal tissue) is indicated. (C) Quantification of endogenous secreted SHH by ELISA assay in A549, H522, and H460 cells culture medium. H460 cell line is presented as a negative control. (D) Quantification of endogenous secreted SHH by ELISA assay in the culture medium of A549 cells transfected with scramble or SHH siRNA. (E) CDON immunofluorescence staining of A549 cells transfected with scramble or CDON siRNA using a CDON-specific antibody (in green) as described in the methods section. Nuclei were stained with Hoechst (in blue). (F) Apoptotic cell death induction as measured by TUNEL staining was quantified in A549 and H522 cells transfected with SHH siRNA alone or together with CDON siRNA. (G) Caspase-9 activity was measured in A549 cells 18 h after transfection with SHH siRNA alone or together with CDON siRNA. (H) *Nude* mice were engrafted with A549 cells by subcutaneous injection of 10 million cells. When the mean tumor volume reached approximately 100 mm^3^, animals were treated twice a week by i.p. injection of scramble or SHH siRNA alone or in combination with CDON siRNA during 4 wk. Mean tumor volume and number of animals for each group are indicated. (I) Representative images of scr siRNA, SHH siRNA, or SHH siRNA+CDON siRNA-treated tumors on day 35. (J) Apoptosis quantification by caspase-3 activity assay on xenografted tumor lysates analyzed after 1 wk of treatment with siRNAs. For (H), error bars indicate s.e.m. Statistical treatment of the data was performed using a two-sided Mann–Whitney test compared to scramble siRNA-treated condition (*p<0.05; ***p<0.001). For (D), (F), (G), and (J), data are means of a minimum of three independent assays. Error bars indicate s.d. Statistical treatment of the data was performed using a two-sided Mann–Whitney test compared to scramble siRNA-transfected condition (*p<0.05).

We first analyzed whether, as described by others (for reviews see [38],[39]), SHH is up-regulated in a sizeable fraction of human cancers. We analyzed the level of SHH mRNA by Q-RT-PCR in colon, lung, and kidney tumors as compared to normal adjacent tissues. As shown in Figure 5AB, SHH was up-regulated in a large fraction of colon and kidney cancers and in a sizeable fraction of lung cancers. According to the dependence receptor paradigm, most of SHH-expressing tumors showed expression of CDON (Figure S4D). We then screened, by Q-RT-PCR, a panel of 59 human tumor cell lines for expression of both SHH and CDON. Of the cell lines tested, 31% showed significant expression of both CDON and SHH (not shown). The A549 and H522 cell lines were selected for their significant expression of CDON mRNA and protein (Figure S4EF). These cell lines both express a secreted form of SHH that could be quantified by ELISA (respectively, 10.0±2.4 pg and 4.0±1.6 pg per mL of culture medium), thus arguing for a secreted/autocrine expression of SHH (Figure 5CD). To investigate the role of this SHH production in regulating CDON-induced apoptosis, SHH expression was reduced by an RNA interference-based strategy. Transfection of both A549 and H522 cell lines with SHH and/or CDON respective siRNAs was associated with a specific and significant decrease in each targeted mRNA (Figure S4F) and protein (Figure 5DE). SHH siRNA transfection induced an increase in apoptosis monitored by an increase in caspase-3 activity and by an increase in TUNEL staining (Figures 5F and S4G). However, co-transfection of a CDON siRNA abrogated SHH siRNA-induced cell death in both cell lines (Figures 5F and S4F). Similar effects were observed with another SHH siRNA and another CDON siRNA (Figure S5AB). A dominant negative mutant of Ptc (Ptc-DN) failed to inhibit SHH siRNA-mediated caspase-3 activity in A549 cells (Figure S5C). In addition, SHH siRNA transfection in A549 cells was associated with caspase-9 activation, which was inhibited when CDON siRNA was co-transfected (Figure 5G). Thus together these data support the view that in SHH/CDON-expressing tumor cells, SHH constitutively inhibits CDON-induced apoptosis.

To assess whether this CDON-mediated cell death upon SHH interference observed *in vitro* could be translated *in vivo*, the A549 cell line was engrafted in *nude* mice. Animals with established tumors were treated twice a week by i.p. injection of either scrambled siRNA or SHH siRNA, alone or in combination with CDON siRNA for 28 d. Such siRNA i.p. injection allowed the detection of the siRNA within the tumor (Figure S5D) and was shown to affect the target gene (Figure S5E and [40]–[43]). As shown in Figures 5HI, treatment with SHH siRNA was associated with tumor growth inhibition as compared to scrambled siRNA-treated mice. As noted *in vitro*, injection of CDON siRNA impaired the SHH siRNA antitumor effect, hence demonstrating that, in this model, the antitumor effect associated with SHH inhibition required CDON. While apoptosis as measured by caspase-3 activity or TUNEL was significantly increased in tumors from SHH siRNA-treated mice, no significant change was observed in terms of cell proliferation (Figures 5J and S5G). As expected, the increase of apoptosis was not observed in tumors from CDON/SHH siRNA-treated mice (Figures 5J and S5G). Moreover, histological analysis of tumors at the end of the treatment revealed a large central area devoid of proliferating tumor cells specifically in SHH siRNA-treated mice but not in SHH/CDON siRNA-treated animals (Figure S5H). Taken together, these data support the view that SHH expression in SHH-high cancer cells is a survival selective advantage that prevents CDON-induced apoptosis.

**Figure 6:**
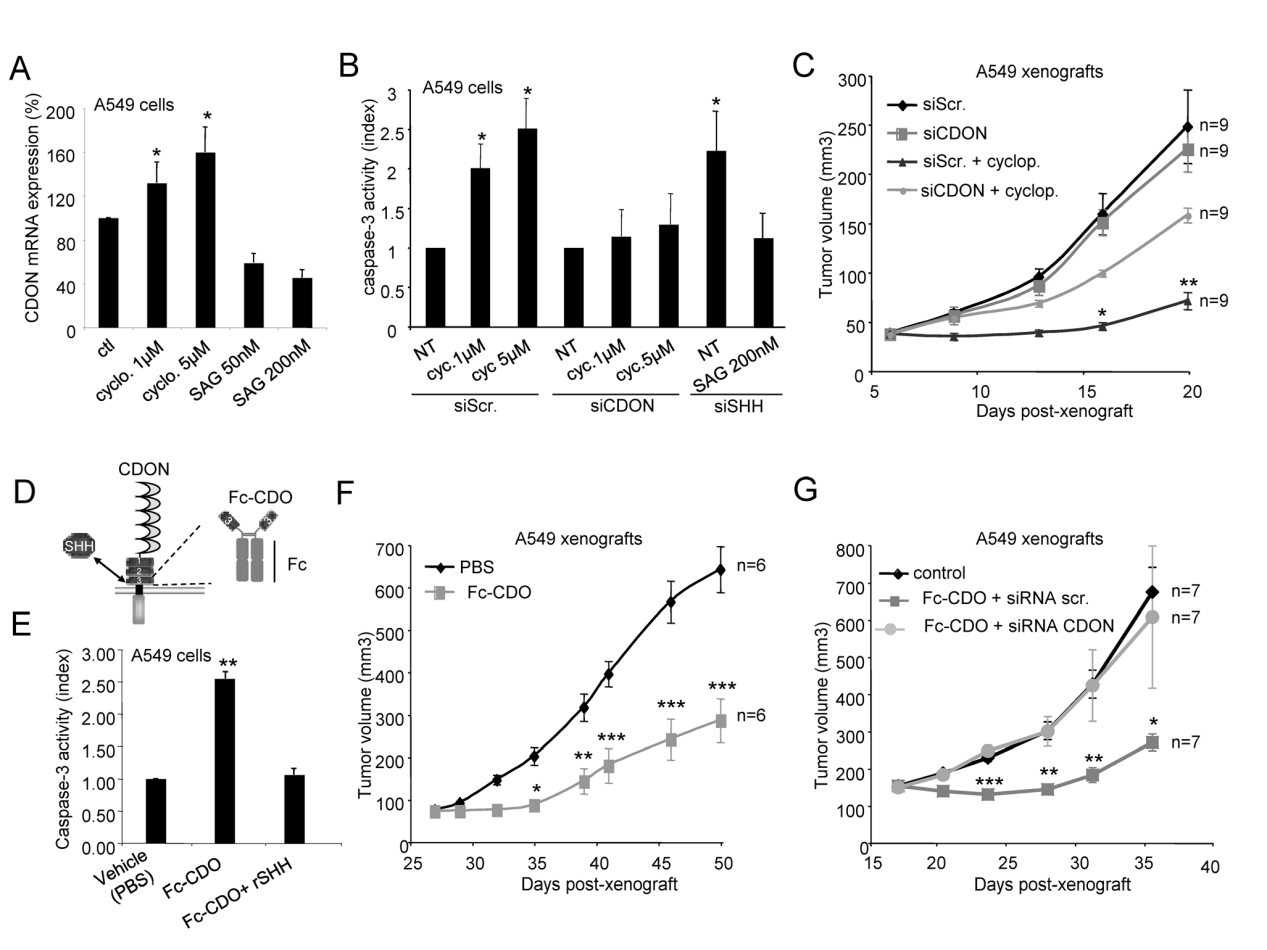
Interference with SHH/CDON interaction as a promising therapeutic strategy. (A) Quantification of CDON mRNA was performed after treatment of A549 cells either with 1 µM or 5 µM cyclopamine (cyclo.) or with 50 nM or 200 nM Smoothened Agonist (SAG). (B) Apoptotic cell death induction as measured by caspase-3 activity was quantified in A549 cells treated with cyclopamine (cyc.) and transfected either with scramble (siScr.) or with CDON siRNA. A549 cells were also treated with SAG and transfected either with siScr or with SHH siRNA. For (A) and (B), data are means of a minimum of three independent assays. Error bars indicate s.d. Statistical treatment of the data was performed using a two-sided Mann–Whitney test compared to scramble siRNA-transfected condition (*p<0.05). (C) *Nude* mice were engrafted with A549 cells by subcutaneous injection of 10 million cells. When the mean tumor volume reached approximately 40 mm^3^, animals were treated daily by intra-tumoral injection of cyclopamine and either scramble or CDON siRNA alone during 13 d. Mean tumor volume and number of animals for each group are indicated (*p<0.05). (D) Schematic representation of the Fc-CDO_3FbnIII_ recombinant protein including the CDON juxtamembrane fibronectin domain associated with a human IgG1 Fc fragment. (E) Apoptotic cell death induction as measured by caspase-3 activity was quantified in A549 cells treated with 2 µg/mL Fc-CDO_3FbnIII_ alone or together with an excess recombinant SHH added in the culture medium. Data are means of a minimum of three independent assays. Error bars indicate s.d. Statistical treatment of the data was performed using a two-sided Mann–Whitney test compared to control condition (*p<0.05). (F) *Nude* mice were engrafted with A549 cells by subcutaneous injection of 10 million cells. When the mean tumor volume reached approximately 100 mm^3^, animals were treated twice a week by i.p. injection of 10 mg/kg Fc-CDO_3FbnIII_ during 3 wk. Mean tumor volume and number of animals for each group are indicated. Error bars indicate s.e.m. Statistical treatment of the data was performed using a two-sided Mann–Whitney test compared to PBS-treated condition (***p<0.001). (G) *Nude* mice were engrafted with A549 cells by subcutaneous injection of 10 million cells. When the mean tumor volume reached approximately 100 mm^3^, animals were treated twice a week by i.p. injection of 20 mg/kg Fc-CDON_3FbnIII_ together with 3 µg siRNA (scramble or CDON siRNA) during 3 wk. Mean tumor volume and number of animals for each group are indicated. Error bars indicate s.e.m. Statistical treatment of the data was performed using a two-sided Mann–Whitney test compared to PBS-treated condition (***p<0.001).

The prevalent hedgehog-related therapeutic strategy assessed so far is the inhibition of the Smo/Gli-1 canonical pathway, which is believed to be activated in some cancers because of Ptc/Smo mutation(s). Smo antagonists such as cyclopamine have shown interesting tumor cell death effects *in vitro* and tumor growth inhibition activity in animal models [11],[12]. Of interest, CDON expression was shown to be down-regulated by the Smo/Gli-1 canonical pathway [13]. We thus investigated whether tumor cell death observed upon Smo antagonists may be somehow related to a CDON up-regulation and to a subsequent increased CDON-mediated cell death. In agreement with Tenzen and collaborators' work, while A549 cells treated with cyclopamine showed an increased level of CDON mRNA, SAG treatment was associated with a decrease in CDON mRNA level (Figures 6A and S6AB). In this setting, cyclopamine treatment was associated with A549 cell death, which was concomitant with CDON expression increase (Figures 6B and S6B). However, this death effect was fully blocked when cells were transfected with CDON siRNA (Figure 6B). Similar results were observed in H522 and MiaPaca-2 cells (not shown). Thus, tumor cell death induced by cyclopamine is, at least in part, due to the up-regulation of CDON expression. We then assessed whether part of the tumor growth inhibition effect of smo-inhibitors such as cyclopamine or GDC-0449 could be explained by an increase in CDON-induced apoptosis *in vivo*. A549 cells were engrafted in *nude* mice and animals were treated by intratumoral injection of cyclopamine (or HBC vehicle) together with scramble siRNA or CDON siRNA for 2 wk. As shown in Figure 6C, while cyclopamine showed a significant tumor growth inhibitory effect, this antitumor effect was in part inhibited when CDON expression was silenced. Similar results were obtained when the Hedgehog inhibitor from Genentech was used instead of cyclopamine (Figure S6C). Together, these data suggest that the Smo antagonists display antitumor efficacy in part because they enhance CDON expression level and consequently CDON-induced apoptosis.

### Early Preclinical Development of an Inhibitor of SHH/CDON Interaction

The *in vitro* and *in vivo* data presented above suggest that interfering with SHH/CDON interaction may be an efficient anticancer strategy in SHH-expressing tumors. We thus developed a drug candidate that is able to inhibit the CDON–SHH interaction. To do so, the third fibronectin domain of CDON, which is known to interact with SHH [44], was fused to a human IgG1 Fc fragment to create a stabilized recombinant protein (see schematic representation in Figure 6D) with adequate pharmacokinetic properties (upon single dose i.v. 20 mg/kg in mouse plasma, terminal half-life: 38 h, %Extrapolated AUC: 24.9, not shown). This Fc-CDON_3FbnIII_ recombinant protein titrates out SHH (not shown). Of interest, Fc-CDON_3FbnIII_ efficiently triggered apoptosis of A549 cells, a process blocked when an excess of recombinant SHH was added (Figure 6E). Similar pro-apoptotic effects of Fc-CDON_3FbnIII_ were obtained with the colorectal HT-29, the pancreatic PANC-1, and the breast MDA-MB436 cancer cell lines, which all express CDON and SHH mRNA (Figure S6DE and not shown).

The *in vivo* activity of Fc-CDON_3FbnIII_ was then assessed against A549, MiaPaca-2, and HT-29 cells engrafted in *nude* mice. As shown in Figure 6F and Figure S6CF, twice a week injection of Fc-CDON_3FbnIII_ (10 mg/kg and 20 mg/kg, respectively) was associated with marked tumor growth inhibition. Moreover, in these settings, co-injection of Fc-CDON_3FbnIII_ together with a siRNA targeting CDON expression completely abrogated Fc-CDON_3FbnIII_ antitumor effect (Figure 6G). Thus, targeting CDON/SHH interaction triggers tumor cell death and tumor growth inhibition by engaging CDON pro-apoptotic activity.

## Discussion

The data presented here suggest that CDON displays all of the characteristics of a dependence receptor: CDON triggers apoptosis in the absence of its ligand SHH, CDON is cleaved at aspartic acid residue(s) by caspase-like protease(s), and CDON triggers caspase-9–dependent apoptosis through a domain exposed by this proteolytic cleavage. These *in vitro* features have been shown to be common to the vast majority of dependence receptors [20]. In addition, we have shown that these traits confer to the pair SHH/CDON a key regulatory role in cancer progression as recently shown for the dependence receptor DCC [23]. If the pro-apoptotic activity of CDON *per se* appears to be fully independent of the classic Hedgehog canonical pathway, CDON expression is dependent on this canonical pathway.

Although the specific mechanism by which CDON triggers apoptosis remains to be dissected, this effect may have important potential as a novel therapeutic anticancer strategy. SHH signaling is indeed a major focus for drug development, since SHH and its downstream signaling have been believed to be frequently deregulated in cancer. We show here that loss of CDON expression in cancers may be a selective advantage for survival in the presence of a limited concentration of SHH in the environment. Conversely, our present data and previous reports support the view that a fraction of tumors from various cancer types shows expression of SHH [3]–[6],[34]–[37],[45]. Even though this observation has been mostly investigated assuming the view of the aberrant activation of SHH–Ptc–Smo–Gli signaling either in tumor cells or more recently in stromal cells [10],[11], considering our results here, it is tempting to revisit this expression of SHH as a mechanism to block CDON-induced apoptosis. Along this line, it is then interesting to consider our results in the light of the recent results of the phase II clinical trials on the Smo antagonist GDC-0449 in colorectal and ovarian cancer: while GDC-0449 clearly demonstrated a benefit in basal cell carcinoma and medullobastoma in which activating mutations of the Ptc–Smo–Gli pathway were shown [46], this drug had no effect on colorectal and ovarian cancer even though a large fraction of the patients included had tumors with SHH expression. According to our data, SHH expression in cancer may not (only) constitutively activate the Ptc–Smo–Gli canonical pathway but rather support survival by blocking CDON apoptotic activity. Moreover, even though in the cell models tested here we have failed to detect the implication of Ptc in the death observed upon silencing of SHH, we may speculate that, as Ptc was also described as a dependence receptor [25], SHH expression may also support survival by blocking Ptc-induced apoptosis in some SHH-high cancers.

The *in vitro* tumor cell death effect and the *in vivo* antitumor effect of SHH interference on SHH-expressing tumor cells support the view that the SHH–CDON interaction is a potential target for drug development. We propose here that a drug interfering with the interaction between SHH and its dependence receptor CDON, such as the presented Fc-CDON_3FbnIII_, could be of potential benefit to the large number of patients suffering from cancers with high SHH levels. Even though further preclinical and clinical testing remains to be performed, the proposed strategy is fundamentally different from the current strategies that aim at antagonizing Smo-mediated signaling.

## Experimental Procedures

### Ethics Statement

Mice were maintained in a specific pathogen-free animal facility, AniCan, with technical help of the Laboratoire des Modèles tumoraux (LMT) and handled in accordance with the institutional guidelines and protocols approved by the animal care and use committee (Comité d'Evaluation Commun au Centre Léon Bérard, à l'Animalerie de transit de l'ENS, au PBES et au laboratoire P4; CECCAP).

The use of all patient tissue specimens was carried out according to French laws and regulations.

### Cell Lines, Transfection Procedures, Reagents

Human embryonic kidney HEK293T, mouse embryo fibroblast NIH3T3, human lung cancer A549, human colon cancer HT-29, human breast cancer MDA-MB436, human pancreatic cancer PANC-1 and MIAPACA2, and the mouse C2C12 myoblast cell lines were cultured in DMEM medium (Gibco, Invitrogen) containing 10% fetal bovine serum. Human H522 lung cancer cell line was cultured in RPMI 1640 Glutamax medium (Gibco, Invitrogen, Inc, Carlsbad, CA) containing 10% fetal bovine serum. Cell lines were transfected using lipofectamine 2000 reagent (Invitrogen) for small interfering RNA (siRNA) or lipofectamine Plus reagent (Invitrogen) for plasmids. Recombinant human SHH was purchased from R&D system (Minneapolis, MN).

### Human Tumors Samples and Biological Annotations

Following patients' consents, surgical human tumors material was immediately frozen. Human colorectal cancer samples (*n* = 45) and kidney cancer samples (*n* = 23) with matched normal tissues were provided by the tumor bank at the Hospices civils de Lyon, fresh tumor tissue being obtained during surgery prior to any systemic therapy. Human breast cancer samples were obtained from the Biological Resource Center of Centre Léon Bérard, Lyon, France.

### Plasmid Constructs and siRNA

Mouse CDON fragments were PCR amplified using pBABE–mCDON–Myc [47] as a template and cloned in pcDNA3.1 vector using pcDNA3.1 Directional TOPO Expression Kit strategy (Invitrogen). A Flag tag was added to each construct. Mouse CDON IC fragments encompass sequences coding for CDON residues Leu^984^ to Thr^1250^. Point mutations Asp (GAT or GAC) to Asn (AAT or AAC) were created using the QuickChange site-directed mutagenesis strategy (Stratagene) using CDON full-length (for cell death assays) or CDON IC (for *in vitro* caspase cleavage assay) constructs as templates. Patched1 construct and dominant negative mutant for Patched1 (pcDNA3.1–PTC1–DN–HA) have been previously described [25]. Dominant negative caspase encoding plasmids (pcDNA3.1–DNcasp8 and pcDNA3.1–DNcasp9) have been previously described [28]. For cell culture use and *in vivo* experiments, human CDON and SHH siRNAs were designed by Santa Cruz (CA) as a pool of three to five target-specific 20–25 nt siRNAs, whereas siCDON.2 and siSHH.2 were purchased from Sigma. For detection of siRNA within the xenografts *in vivo*, biotinylated SHH or scramble siRNA were purchased from Sigma.

### *In Vitro* Translation and Caspase-3 Cleavage Assay

Plasmids pcDNA3.1–CDON-IC with different point mutations were transcribed using T7 polymerase and then translated using the TNT system (Promega) in the presence of 50 µCi [^35^S]methionine (Perkin Elmer) for 3 h at 30°C. Translation products were incubated for 2 h in 20 mM PIPES pH 7.2, 100 mM NaCl, 1% Chaps, 10% sucrose, 10 mM dithiothreitol, and 0.1 mM EDTA, at 37°C in the presence of purified active caspase-3 kindly provided by G. Salvesen. Samples were loaded on a 14% Tris-Glycine acrylamide gel (Invitrogen).

For the detection of endogenous CDON caspase cleavage fragment, four E12.5 mouse embryonic spinal cords (SCs) were dissected and incubated at 37°C in Hanks' balanced salt solution (HBSS, Invitrogen) for 6 h with the general caspase inhibitor z-VAD-fmk or DMSO as a control. SCs were then lysed in the presence of the general caspase inhibitor z-VAD-fmk or DMSO, and immunoblot was performed on these lysates using a CDON C-terminal domain-specific antibody (Santa Cruz).

### Cell Death Assays

The 1.8×10^5^ cells were grown in serum-poor medium and transfected with plasmids using Lipofectamine Plus Reagent (invitrogen) or with siRNAs using Lipofectamine 2000 (Invitrogen). Cell death was analyzed using trypan blue staining procedures as previously described [18], 24 h after transfection. The extent of cell death is presented as the percentage of trypan blue-positive cells in the different cell populations. Apoptosis was monitored by measuring caspase-3 activity as described previously [18] using caspase-3/CPP32 Fluorimetric Assay Kit (Gentaur Biovision, Brussel, Belgium) 24 h after transfection. For detection of DNA fragmentation, treated cells were cytospun 48 h after transfection, and Terminal deoxynucleotidyl transferase mediated dUTP-biotin Nick End Labeling (TUNEL) was performed with 300 U/mL TUNEL enzyme (300 U/mL) and 6 µM biotinylated dUTP (Roche Diagnostics, Meylan, France), as previously described [48]. Caspase-9 activity was assessed using Caspase-Glo 9 Assay Systems (Promega) according to the manufacturer's protocol.

### Quantitative RT-PCR

To assay SHH and CDON expression in human tumor and healthy tissues and in human cell lines, total RNA was extracted using the Nucleospin RNAII kit (Macherey–Nagel) and 1 µg was reverse-transcribed using the iScript cDNA Synthesis kit (BioRad). Real-time quantitative RT-PCR was performed on a LightCycler 2.0 apparatus (Roche) using either the Light Cycler FastStart DNA Master SYBERGreen I kit (Roche) for CDON and LightCycler TaqMan Master kit (Roche) for SHH. Reaction conditions for all optimal amplifications, as well as primer selection were determined as already described. The ubiquitously expressed human *HPRT* gene was used as an internal control for neuroblastoma, lung, and kidney samples, whereas *PGK* and *HMBS* housekeeping genes were, respectively, selected for colon and breast samples. The sequences of the primers are available upon request.

### Dot Blot Analysis of CDON Expression in Human Tissues

*CDON* gene expression in human tumor and normal paired tissues was monitored by using the Cancer Profiling Array (CLONTECH) following the manufacturer's suggested procedure. CDON probe was prepared by using Amersham Megaprime DNA Labeling System (GE Healthcare) with human full-length CDON cDNA as template. The following primers were used: 5′-GCATCTCGTCCTTATCAAGTGG-3′ and 5′-TATGGTATTCTGCTGGCGATTC-3′. The dot blot was quantified by using Quantity one 4.6.1 software (Biorad). CDON loss of expression was defined by a fold change (normal versus tumor) greater than 2.

### Immunohistochemistry, Co-Immunoprecipitation, and Immunoblotting Analysis

For immunohistochemistry on cells, 6.5×10^4^ cells were grown on coverslips. For immunohistochemistry on tumors, samples previously fixed in 4% paraformaldehyde were embedded in 7.5% gelatin—0.12 M sucrose and 20 µm sections were performed. Slides were then incubated at room temperature for 2 h with a primary antibody recognizing the human CDON (1∶200, R&D systems, Minneapolis, MN), the FlagM2 tag (1/400, Sigma), or the HA tag (1/500, Sigma). After rinsing in phosphate buffer saline, the slides were incubated with an Alexa-488-Donkey or Cy3-Donkey anti-goat antibody (Molecular Probes), or Cy5-donkey anti-mouse/anti-rabbit/anti-goat antibodies (Jackson ImmunoResearch, Suffolk, UK) (Molecular Probes), respectively. Nuclei were visualized with Hoechst staining. Fluorescence imaging was performed with AxioVision Release 4.6 software. Antibody for CDON immunohistochemistry was validated after transfection of siRNA silencing CDON expression.

Co-immunprecipitations and immunoblots were performed as already described using anti-CDON (1/2,000, R&D Systems), anti-FlagM2 (1/5,000, Sigma), anti-HA (1∶7,500, Sigma), anti-caspase-9 (1∶1,000, Cell Signaling), or anti-β-actin (1∶1,000, Chemicon) primary antibodies.

### SHH ELISA Assay

For SHH determination in cell culture supernatants, white 96-well plates (CORNING) were coated with a monoclonal anti-mouse SHH N-term peptide antibody (MAB4641—R&D systems), blocked with bovine serum albumin, incubated with the samples, followed by detection of SHH using a biotinylated anti-mouse SHH antibody (BAM4641—R&D systems), a streptavidin-peroxidase polymer (S2438—Sigma), and a chemiluminescent substrate (Pierce ECL Western Blotting Substrate). The luminescence was read on a Tecan Infinite F-500 luminometer.

### Mice

APC^+/1638N^ mice (in C57BL/6 background; a gift from R. Fodde) were mated with mice heterozygous CDON^+/−^ in 129SVpass background. Double heterozygous CDON^+/−^ APC^+/1638N^ mice of the offspring were interbred to generate knockout mice for the *CDON* gene and heterozygous for APC [33]. Even though no change in APC-dependent intestinal tumor propension has been described between C57BL/6 and 129SV pass [33], littermates from CDON^+/−^APC^+/1638N^ intercrosses were analyzed in a blinded manner to minimalize variation due to background heterogeneity. Routine genotype analysis of mice was performed by PCR assay on DNA purified from fingers biopsies (Red extract kit, Sigma Aldrich). Littermate mice were euthanized at 6 mo. Tumors were detected in different places of the intestines including the colon. In all cases, intestines were removed and examined for the presence of neoplasia. Tumors were resected, formalin fixed, and paraffin embedded. We stained 3-µm-thick sections with haematoxylin–eosin–saffron (HES). Histological classification and grading of neoplastic lesions was performed in a blinded fashion and according to standard procedures.

Quantification of apoptosis was performed in blind on 12 different fields of size-matched high-grade adenomas sections stained with HES from three different mice of each genotype (CDON^+/+^ and CDON^−/−^). Pyknotic cells show retracted and hyperchromatic nuclei.

### SHH Inhibition in A549-Engrafted *Nude* Mice

Seven-week-old (20–22 g body weight) female athymic nu/nu mice were obtained from Charles River animal facility. The mice were housed in sterilized filter-topped cages and maintained in a pathogen-free animal facility. A549 and HT-29 cells were implanted by s.c. injection of 10^7^ cells or 5.10^6^ cells, respectively, in 200 µL of PBS into the right flank of the mice. Once tumors were established (V≈100 mm^3^), mice were treated with scramble siRNA, SHH siRNA, and/or CDON siRNA by i.p. injection of 6 µg total siRNA during 4 wk, twice a week, and/or with 10 or 20 mg/kg Fc-CDON_3FbnIII_ (using PBS-glycerol as a control) during 3 wk twice a week. Tumor sizes were measured with a caliper. The tumor volume was calculated with the formula v = 0.5×(length×width^2^). At the end of the treatment, tumors were harvested, weighted, and were embedded in 7.5% gelatin—0.12 M sucrose and sectioned into 20 µm slices. Tumors' histology was studied after Hematoxylin–PhloxinB–Saffron staining of tumor slides. To measure apoptotic cell death, some tumors were harvested after 1 wk of treatment, immediately frozen, and then mechanically lysed in caspase-3 activity lysis buffer. Then, caspase-3 activity was quantified using caspase-3/CPP32 Fluorimetric Assay Kit (Gentaur Biovision).

For the cyclopamine/siRNA co-treatment experiment, cyclopamine was prepared as a complex with 2-hydroxypropyl-β-cyclodextrin (HBC, Sigma) (1 mg cyclopamine per milliliter of 45% HBC in sterile PBS stirred for 60 min at 65°C [49]). Once tumors reached approximately 40 mm^3^, 3 µg of scramble or CDON siRNA were then added to 100 µL of cyclopamine-HBC or HBC alone, and the mix was injected intra/peri-tumorally daily during 13 d. For the GDC-0449 (Hh inhibitor)/siRNA treatment, when MiaPaca-2 cells xenografts reached 100–125 mm^3^, animals were separated into groups with similarly sized tumors and drug administration was initiated. GDC-0449 was daily administered at 20 mg/kg during 15 d. Tumor size was quantified by external calipers.

## Supporting Information

Figure S1 **CDON, but not BOC, induces apoptosis *in vitro*, a mechanism dependent of CDON caspase cleavage.** (A) Schematic representation of BOC protein structural domains. (B) Cell death induction in HEK293T cells was quantified by trypan blue exclusion assay after transfection with mock or BOC-expressing constructs and treatment with recombinant SHH added in the cell culture medium. (C) Cell death induction in NIH-3T3 cells was quantified by caspase-3 activity assay after transfection with CDON alone or with recombinant SHH or treatment with SAG. (D) Apoptotic cell death induction as measured by caspase-3 activity was quantified in HEK293T cells transfected with full-length DCC alone or with recombinant SHH (rSHH) added in excess in the culture medium. Upper panel shows detection of DCC-HA protein by immunoblot using anti-HA antibody. (E) Apoptotic cell death induction as measured by caspase-3 activity was quantified in HEK293T cells transfected with full-length CDON alone or with recombinant SHH (SHH) or Desert hedgehog (dhh) added in excess in the culture medium. (F) Detection of endogenous full-length CDON and CDON fragment released by its intracellular cleavage by Western blot in E12.5 mouse embryonic spinal cord (SC) lysates in the presence of the general caspase inhibitor z-VAD-fmk or DMSO as a control, using a CDON C-terminal domain-specific antibody. For (B–D) and (E), data are means of a minimum of three independent assays. Error bars indicate s.d. Statistical treatment of the data was performed using a two-sided Mann–Whitney test compared to control condition (*p<0.05; **p<0.01).
(TIFF)

Figure S2 **Characterization of CDON pro-apoptotic activity. (A) Staining of HEK293T transfected with a mouse CDON 1154–1250 fused with GFP expression plasmid.** CDON 1154–1250 (green) and nuclei (Hoechst in blue) staining are shown. (B) Apoptotic cell death induction as measured by caspase-3 activity was quantified in HEK293T cells transfected with constructs encoding CDON or CDON hypothetical fragment resulting from its cleavage by caspase at D1153 (CDON 1–1153) and treated or not with 900 ng/mL recombinant SHH added in the culture medium. (C) C2C12 cell lysates were subjected to immunoprecipitation with a CDON-specific antibody. Endogenous CDON and endogenous caspase-9 proteins were detected by Western blot in immunoprecipitated and input fractions. (D) Apoptotic cell death induction as measured by caspase-3 activity was quantified in HEK293T cells transfected with a construct encoding CDON together with caspase-9 dominant negative (DN-C9) or caspase-8 dominant negative (DN-C8) constructs. Upper panel shows detection of CDON (α-CDON), DN-C9 (α-Flag), and DN-C8 (α-HA) by immunoblot. For (B) and (D) error bars indicate s.d. Statistical treatment of the data was performed using a two-sided Mann–Whitney test compared to control condition (*p<0.05).
(TIFF)

Figure S3 **CDON-induced apoptosis is independent of the canonical SHH pathway.** (A) Gli1 transcriptional activity was measured in NIH3T3 cells transfected with a Gli1-responsive element cloned upstream of the firefly luciferase reporter gene. Cells were co-transfected with constructs encoding Gli1 and/or different CDON fragments, and/or treated with 50 nM Smoothened Agonist (SAG). Firefly luciferase activity was indexed on renilla luciferase activity used as an internal transfection control. (B) Apoptotic cell death induction as measured by caspase-3 activity was quantified in HEK293T cells transfected with a construct encoding CDON alone or together with Gli1, or after treatment with 200 nM SAG. (C) Apoptotic cell death induction as measured by caspase-3 activity was quantified in HEK293T cells transfected with a construct encoding CDON, Ptc, or Ptc-DN. (D) Apoptotic cell death induction as measured by caspase-3 activity was quantified in HEK293T cells transfected with a construct encoding CDON, Ptc, or CDON-DN (CDON-IC–D1153N/D1164N). (E) Apoptotic cell death induction as measured by caspase-3 activity was quantified in HEK293T cells transfected with a construct encoding CDON, Ptc, and siRNA silencing CDON and Ptc. For (A–E) data are means of at least three independent assays. Error bars indicate s.d. Statistical treatment of the data was performed using a two-sided Mann–Whitney test compared to mock-transfected condition (*p<0.05; ** *p*<0.01).
(TIFF)

Figure S4 **SHH is an essential protein inhibiting CDON-induced cell death in cancer cells.** (A) Quantification of CDON expression by Q-RT-PCR in a panel of 45 human colorectal tumors and paired normal tissues. Data are presented as CDON expression between tumor (T) and the mean value for normal (N) tissues. (B) Quantification of CDON expression by dot blot array analysis in a panel of 113 human tumors and their paired normal tissues. For each type of tissue, the percentage of tumors showing loss of CDON expression is indicated. Loss of CDON expression in the tumor is considered when a more than 2-fold decrease of expression is observed as compared to the normal tissue. (C) Quantification of active caspase-3 in high-grade adenomas in CDON^+/+^ APC^+/1638N^ mice compared to CDON^−/−^ APC^+/1638N^ mice. (D) In the tumors analyzed in Figure 5B for SHH up-regulation, CDO expression was analysed by Q-RT-PCR. (E) CDON immunofluorescence staining of A549 and H522 cells using anti-CDON antibody to reveal CDON endogenous expression. (Inset) Immunostaining without primary antibody is presented as a control. (F) Quantification of SHH and CDON expression by Q-RT-PCR was performed to check the efficiency and specificity of SHH and CDON siRNAs 24 h after transfection of A549 cells. (G) Apoptotic cell death induction as measured by caspase-3 activity was quantified in A549 and H522 cells transfected with SHH siRNA alone or together with CDON siRNA (*p<0.05). Data are means of a minimum of three independent assays. Error bars indicate s.d. Statistical treatment of the data was performed using a two-sided Mann–Whitney test compared to control condition (*p,0.05).
(TIFF)

Figure S5 ***In vitro* and *in vivo* evaluation of CDON-induced apoptosis in SHH-expressing tumor cell lines.** (A) Quantification of SHH and CDON expression by Q-RT-PCR was performed to check the efficiency and specificity of two other CDON and SHH siRNAs (respectively, siCDON.2 and siSHH.2) 24 h after transfection of A549 cells. (B) Apoptotic cell death induction as measured by caspase-3 activity was quantified in A549 cells transfected with two different SHH siRNAs (siSHH and siSHH.2) together with scramble siRNA or two different CDON siRNAs (siCDON and siCDON.2). Each SHH siRNA specificity was assessed by adding recombinant SHH (+rSHH) in the culture medium. (C) Apoptotic cell death was quantified by caspase-3 assay in A549 cells transfected with SHH siRNA alone or together with Ptc-DN construct. For (B–C), data are means of a minimum of three independent assays. Error bars indicate s.d. Statistical treatment of the data was performed using a two-sided Mann–Whitney test compared to control transfected condition (*p<0.05). (D) A biotinylated SHH siRNA (siSHH-[btn]) was injected i.p. in *nude* mice bearing A549 xenografts. Thirty minutes after injection, tumors were harvested and siSHH-[btn] was detected within the tumor with Cy3-Streptavidine (in orange). Nuclei were stained with Hoechst (in blue). PBS intra-peritoneal injection was used as a control. (E) Quantification of CDON and SHH mRNA by Q-RT-PCR in A549 xenografts 16 h after i.p. injection of scramble (siScr.), CDON (siCDON), or SHH (siSHH) siRNAs. mRNA expression was analyzed in two different xenografts for each siRNA (.1 and .2). HPRT was used as a housekeeping gene. (F) Mean tumor mass of scr siRNA, SHH siRNA, or SHH siRNA+CDON siRNA-treated tumors on day 46, at the end of treatment. (G) Quantification of apoptosis and proliferation in tumor sections. The apoptotic ratio was calculated as the percentage of TUNEL positive cells counted in 20 random fields using TUNEL staining on paraffin-embedded tumor sections (*p<0.05). The proliferation index was determined by counting the Ki67 positives cells. (H) Xenograft histology was analyzed by Hematoxylin–PhloxinB–Safran (HPS) staining on tumors harvested at the end of the treatment. Note that SHH siRNA-treated xenografts are characterized by a vast central area devoid of tumor cells (white arrows). Scale bar, 1 mm.
(TIFF)

Figure S6 **Interference with SHH/CDON interaction in different human cell lines.** (A) Quantification of SHH mRNA was performed after treatment of A549 cells either with 5 µM cyclopamine (cyclo.) or with 200 nM Smoothened Agonist (SAG). (B) Time course of CDON-induced cell death upon cyclopamine treatment (5 µM) measured by caspase-3 activation and correlated with CDON expression quantified by Q-RT-PCR. Error bars indicate s.d. (C) *Nude* mice were engrafted with MiaPaca-2 cells by subcutaneous injection of 5 million cells. When the mean tumor volume reached approximately 150 mm^3^, animals were treated with GDC-0449 (20 mg/kg) and twice a week by i.p. injection of 20 mg/kg Fc-CDO_3FbnIII_ during 2 wk. Mean tumor volume and number of animals for each group are indicated. Error bars indicate s.e.m. Statistical treatment of the data was performed using a two-sided Mann–Whitney test compared to PBS-treated condition (*p<0.05). (D) Quantification of CDON and SHH mRNA expression by Q-RT-PCR in HT-29 colon, MDA-MB436 breast, and PANC-1 pancreatic cancer cell lines. PGK expression was used as a housekeeping gene. (E) Apoptotic cell death induction as measured by caspase-3 activity was quantified in PANC-1 and MDA-MB436 cells treated with 10 µg/mL Fc-CDO_3FbnIII_ alone or together with an excess recombinant SHH added in the culture medium. Data are means of a minimum of three independent assays. Error bars indicate s.d. Statistical treatment of the data was performed using a two-sided Mann–Whitney test compared to control condition (*p<0.05). (F) *Nude* mice were engrafted with HT-29 cells by subcutaneous injection of 5 million cells. When the mean tumor volume reached approximately 100 mm^3^, animals were treated twice a week by i.p. injection of 20 mg/kg Fc-CDO_3FbnIII_ during 2 wk. Mean tumor volume and number of animals for each group are indicated. Error bars indicate s.e.m. Statistical treatment of the data was performed using a two-sided Mann–Whitney test compared to PBS-treated condition (*p<0.05).
(TIFF)

## Abbreviations

ADK: adenocarcinoma
BOC: brother of CDON
CDON: cell-adhesion molecule-related/down-regulated by Oncogenes
DCC: deleted in colorectal cancer
Gli: Glioma-associated family of transcription factors
Ptc: Patched 1
SHH: Sonic Hedgehog
Smo: Smoothened
TUNEL: terminal deoxynucleotidyl transferase–mediated deoxyUridine triphosphate Nick End Labeling
