# Supplementary figures and images for: Sonic Hedgehog Promotes Tumor Cell Survival by Inhibiting CDON Pro-Apoptotic Activity
Source: PLoS Biol. 2013 Aug 6;11(8):e1001623. doi: 10.1371/journal.pbio.1001623 (PMC3735457; doi:10.1371/journal.pbio.1001623)

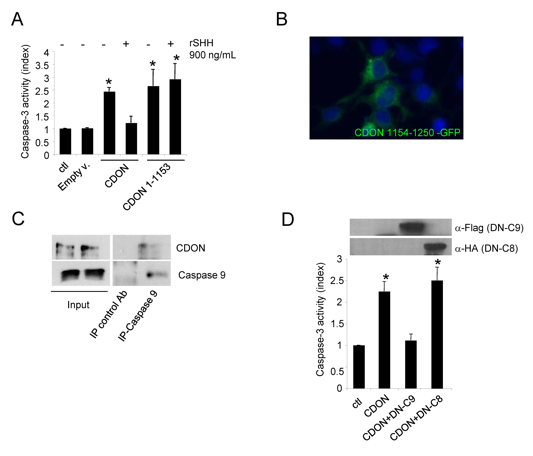

Supplement: Figure S2 — Characterization of CDON pro-apoptotic activity. (A) Staining of HEK293T transfected with a mouse CDON 1154–1250 fused with GFP expression plasmid. CDON 1154–1250 (green) and nuclei (Hoechst in blue) staining are shown. (B) Apoptotic cell death induction as measured by caspase-3 activity was quantified in HEK293T cells transfected with constructs encoding CDON or CDON hypothetical fragment resulting from its cleavage by caspase at D1153 (CDON 1–1153) and treated or not with 900 ng/mL recombinant SHH added in the culture medium. (C) C2C12 cell lysates were subjected to immunoprecipitation with a CDON-specific antibody. Endogenous CDON and endogenous caspase-9 proteins were detected by Western blot in immunoprecipitated and input fractions. (D) Apoptotic cell death induction as measured by caspase-3 activity was quantified in HEK293T cells transfected with a construct encoding CDON together with caspase-9 dominant negative (DN-C9) or caspase-8 dominant negative (DN-C8) constructs. Upper panel shows detection of CDON (α-CDON), DN-C9 (α-Flag), and DN-C8 (α-HA) by immunoblot. For (B) and (D) error bars indicate s.d. Statistical treatment of the data was performed using a two-sided Mann–Whitney test compared to control condition (*p<0.05). (TIFF) [file pbio.1001623.s002.tiff]

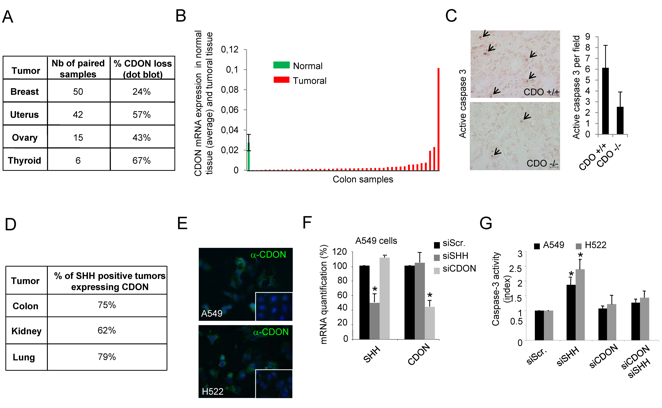

Supplement: Figure S4 — SHH is an essential protein inhibiting CDON-induced cell death in cancer cells. (A) Quantification of CDON expression by Q-RT-PCR in a panel of 45 human colorectal tumors and paired normal tissues. Data are presented as CDON expression between tumor (T) and the mean value for normal (N) tissues. (B) Quantification of CDON expression by dot blot array analysis in a panel of 113 human tumors and their paired normal tissues. For each type of tissue, the percentage of tumors showing loss of CDON expression is indicated. Loss of CDON expression in the tumor is considered when a more than 2-fold decrease of expression is observed as compared to the normal tissue. (C) Quantification of active caspase-3 in high-grade adenomas in CDON+/+ APC+/1638N mice compared to CDON−/− APC+/1638N mice. (D) In the tumors analyzed in Figure 5B for SHH up-regulation, CDO expression was analysed by Q-RT-PCR. (E) CDON immunofluorescence staining of A549 and H522 cells using anti-CDON antibody to reveal CDON endogenous expression. (Inset) Immunostaining without primary antibody is presented as a control. (F) Quantification of SHH and CDON expression by Q-RT-PCR was performed to check the efficiency and specificity of SHH and CDON siRNAs 24 h after transfection of A549 cells. (G) Apoptotic cell death induction as measured by caspase-3 activity was quantified in A549 and H522 cells transfected with SHH siRNA alone or together with CDON siRNA (*p<0.05). Data are means of a minimum of three independent assays. Error bars indicate s.d. Statistical treatment of the data was performed using a two-sided Mann–Whitney test compared to control condition (*p,0.05). (TIFF) [file pbio.1001623.s004.tiff]

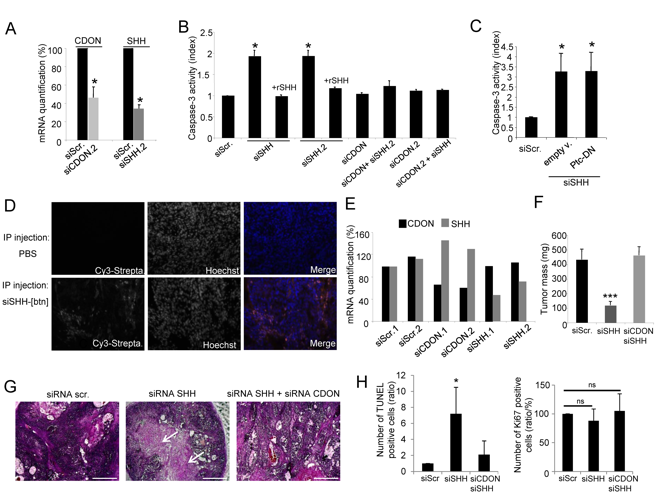

Supplement: Figure S5 — In vitro and in vivo evaluation of CDON-induced apoptosis in SHH-expressing tumor cell lines. (A) Quantification of SHH and CDON expression by Q-RT-PCR was performed to check the efficiency and specificity of two other CDON and SHH siRNAs (respectively, siCDON.2 and siSHH.2) 24 h after transfection of A549 cells. (B) Apoptotic cell death induction as measured by caspase-3 activity was quantified in A549 cells transfected with two different SHH siRNAs (siSHH and siSHH.2) together with scramble siRNA or two different CDON siRNAs (siCDON and siCDON.2). Each SHH siRNA specificity was assessed by adding recombinant SHH (+rSHH) in the culture medium. (C) Apoptotic cell death was quantified by caspase-3 assay in A549 cells transfected with SHH siRNA alone or together with Ptc-DN construct. For (B–C), data are means of a minimum of three independent assays. Error bars indicate s.d. Statistical treatment of the data was performed using a two-sided Mann–Whitney test compared to control transfected condition (*p<0.05). (D) A biotinylated SHH siRNA (siSHH-[btn]) was injected i.p. in nude mice bearing A549 xenografts. Thirty minutes after injection, tumors were harvested and siSHH-[btn] was detected within the tumor with Cy3-Streptavidine (in orange). Nuclei were stained with Hoechst (in blue). PBS intra-peritoneal injection was used as a control. (E) Quantification of CDON and SHH mRNA by Q-RT-PCR in A549 xenografts 16 h after i.p. injection of scramble (siScr.), CDON (siCDON), or SHH (siSHH) siRNAs. mRNA expression was analyzed in two different xenografts for each siRNA (.1 and .2). HPRT was used as a housekeeping gene. (F) Mean tumor mass of scr siRNA, SHH siRNA, or SHH siRNA+CDON siRNA-treated tumors on day 46, at the end of treatment. (G) Quantification of apoptosis and proliferation in tumor sections. The apoptotic ratio was calculated as the percentage of TUNEL positive cells counted in 20 random fields using TUNEL staining on paraffin-embedded tumor sections (*p<0.05). T [file pbio.1001623.s005.tiff]
